# CpG-oligodeoxynucleotides exert remarkable antitumor activity against diffuse malignant peritoneal mesothelioma orthotopic xenografts

**DOI:** 10.1186/s12967-016-0781-4

**Published:** 2016-01-25

**Authors:** Michelandrea De Cesare, Lucia Sfondrini, Marzia Pennati, Cinzia De Marco, Valentina Motta, Elda Tagliabue, Marcello Deraco, Andrea Balsari, Nadia Zaffaroni

**Affiliations:** Molecular Pharmacology Unit, Department of Experimental Oncology and Molecular Medicine, Fondazione IRCCS Istituto Nazionale dei Tumori, Milan, Italy; Dipartimento di Scienze Biomediche per la Salute, Università degli Studi di Milano, Milan, Italy; Biomarker Unit, Department of Experimental Oncology and Molecular Medicine, Fondazione IRCCS Istituto Nazionale dei Tumori, Milan, Italy; Molecular Pathology Unit, Department of Laboratory Medicine, Ospedale Cà Granda Niguarda, Milan, Italy; Molecular Targeting Unit, Department of Experimental Oncology and Molecular Medicine, Fondazione IRCCS Istituto Nazionale dei Tumori, Milan, Italy; Peritoneal Surface Malignancy Program, Department of Surgery, Fondazione IRCCS Istituto Nazionale dei Tumori, Milan, Italy

**Keywords:** CpG-oligodeoxynucleotides, Toll-like receptors, Diffuse malignant peritoneal mesothelioma, Orthotopic xenografts

## Abstract

**Background:**

Diffuse malignant peritoneal mesothelioma (DMPM) is a rare and locally aggressive disease. DMPM prognosis is dismal, mainly due to the lack of effective treatment options and the development of new therapeutic strategies is urgently needed. In this context, novel immunotherapy approaches can be explored in an attempt to improve DMPM patients’ survival.

**Methods:**

We tested the efficacy of CpG-oligodeoxynucleotides (CpG-ODN), synthetic DNA sequences recognized by Toll-like receptor 9 and able to induce innate/adaptive immune response, in two DMPM orthotopic xenografts (MesoII and STO), which properly recapitulate the dissemination pattern of the disease in the peritoneal cavity. Severe combined immunodeficiency mice carrying DMPM xenografts were treated at different stages of tumor development with i.p. delivered CpG-ODN1826 for 4 weeks. CpG-ODN1826-induced modulation in the composition of peritoneal immune infiltrate was assessed by flow cytometry.

**Results:**

When administered to early-stage tumors (i.e., 4 days after i.p. DMPM cell injection in mice), the agent exhibited impressive efficacy against MesoII by completely inhibiting tumor take and ascites development (no evidence of tumor masses and ascites in 6/6 mice at necropsy), and also impaired STO tumor take and growth (4/6 tumor-free mice; i.p. tumor masses reduced by 94 % in the 2 remaining mice, *P* = 0.00005). Interestingly, when tested against late-stage STO tumors (i.e., 11 days after i.p. DMPM cell injection in mice), CpG-ODN1826 was still able to reduce the growth of i.p. tumor masses by 66 % (*P* = 0.0009). Peritoneal washings of tumor-bearing mice revealed a strong increase of macrophage infiltration together with a decrease in the presence of B-1 cells and a reduced IgM concentration after CpG-ODN1826 treatment.

**Conclusions:**

Our results indicate that locally administered CpG-ODN1826 is able to markedly affect the growth of both early- and late-stage DMPM orthotopic xenografts in the absence of severe side effects, and suggest a possible clinical role for the agent in the therapy of DMPM.

## Background

Diffuse malignant peritoneal mesothelioma (DMPM) is an uncommon malignancy that develops from the mesothelial cells lining the peritoneal cavity and accounts for 10–20 % of all mesotheliomas [1]. Although locally invasive rather than metastatic, DMPM is a rapidly fatal disease. Standard therapy with palliative surgery and systemic or intra-peritoneal (i.p.) chemotherapy is associated with a median survival of about 1 year [2]. Although in recent years an integrated approach that combines cytoreductive surgery (CRS) with perioperative hyperthermic i.p. chemotherapy (HIPEC) has improved median survival to 4–5 years [3], approximately half of treated patients experience relapse [4]. Thus, it is mandatory to develop novel strategies to optimize the management of recurrent DMPM patients and to offer valid alternative therapeutic options to patients who cannot undergo CRS + HIPEC due to advanced stage of disease. In this context, novel immunotherapy approaches can be explored in an attempt to improve DMPM patients’ survival.

Toll-like receptors (TLR), which are critically important in detecting pathogens, are potent activators of immune response under consideration for cancer immunotherapy [5, 6]. One of the most promising targets is TLR9, which is activated by synthetic oligodeoxynucleotides (ODN) containing unmethylated CpG motifs (CpG-ODN). Local delivery of CpG-ODN has been explored as a novel immunotherapy approach for cancers suitable for drug injection at the tumor site [5].

Immune cells directly or indirectly activated by CpG-ODN treatment predominantly belong to the innate immune system, such as natural killer (NK) cells, macrophages, neutrophils, monocytes and dendritic cells. Most of these cells require a local activation to exert their effector activity, unlike adaptive immune cells which can reach the target wherever they are activated, and need to be repeatedly stimulated, since their activity is generally reduced rapidly after mediating their effector functions [7].

The peritoneal cavity is characterized by a massive presence of innate immune cells as active biosensors against invading pathogens. These cells may provide a powerful tool for eradicating DMPM, since their antitumor activity can be stimulated by repeated local administrations of CpG-ODN. Here, we evaluated the efficacy of i.p. CpG-ODN1826 against two DMPM orthotopic xenograft models in severe combined immunodeficiency (SCID) mice. The experimental results show that the agent significantly impaired take and growth of both DMPM models.

## Methods

### Cell lines

DMPM cell lines (MesoII and STO), which were established from surgical specimens of patients who underwent surgery at the Fondazione IRCCS Istituto Nazionale dei Tumori (INT) of Milan, were cultured as previously reported [8]. Cell lines were tested fortnightly for the absence of Mycoplasma and periodically monitored for DNA profile of short tandem repeats by the AmpFISTR Identifiler PCR amplification kit (Applied Biosystems, Carlsbad, CA, USA). Both cell lines were last tested in December 2014.

### In vivo studies

The in vivo antitumor activity of purified, phosphorothioated CpG-ODN1826 (5′-TCCATGACGTTCCTGACGTT-3′; TriLinK Biotechnologies, San Diego, CA, USA) was assessed on DMPM cells orthotopically xenotransplanted into 8–10 weeks-old female SCID mice (Charles River, Calco, Italy). Experiments were approved by the Ethics Committee for Animal Experimentation of INT, according to reported guidelines [9]. The origin of orthotopic xenografts was authenticated through microsatellite analysis by the AmpFISTR Identifiler PCR Amplification Kit.

**Fig. 1:**
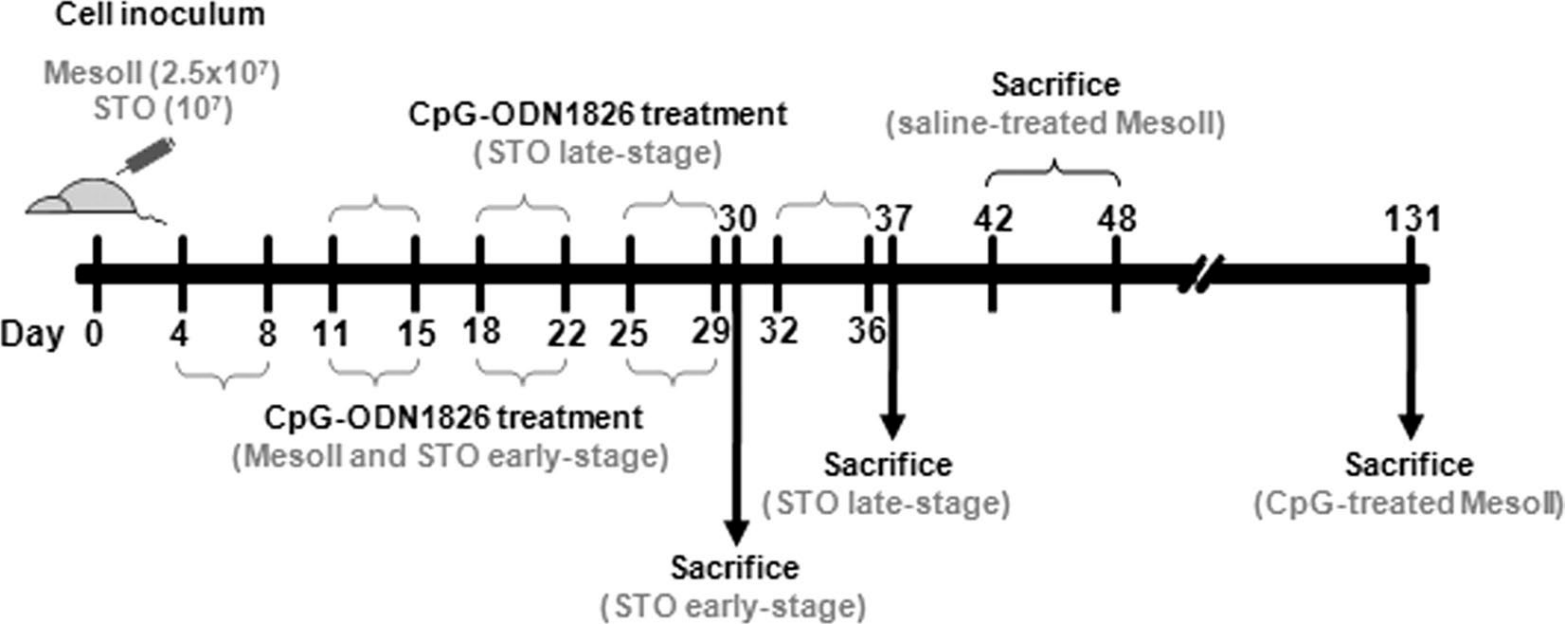
Schematic experimental timeline of DMPM orthotopic model generation, administration of CpG-ODN1826, and sacrifice of mice. Orthotopic models were generated by injecting exponentially growing MesoII and STO cells into the peritoneum of SCID mice. The day after cell injection, mice were randomized to receive saline or CpG-ODN1826 (20 µg/mouse, qdx5/wx4w): CpG-ODN1826 treatment started 4 days (STO and MesoII early-stage tumors) or 11 days (STO late-stage tumors) after cell injection. MesoII cell-injected mice were sacrificed as ascites was evident; STO cell-injected mice were sacrificed the day after the last CpG-ODN1826 treatment

Orthotopic models were generated by injecting 2.5 × 10^7^ and 1 × 10^7^ exponentially growing MesoII and STO cells, respectively, suspended in 500 µl saline in the peritoneum of SCID mice. The day after cell injection, mice were randomized (6–10 mice/group) to receive saline or CpG-ODN1826 delivered i.p. at 20 µg/mouse, every day for five days a week for four weeks (qdx5/wx4w). CpG-ODN1826 treatment started 4 days (STO and MesoII early-stage tumors) or 11 days (STO late-stage tumors) after cell injection (Fig. 1).

MesoII cell-injected mice developed hemorrhagic ascites in >40 days. The animals were inspected and weighed daily, and sacrificed as ascites became evident (Fig. 1). The ratio of the median day of ascites onset in treated over control mice (T/C) × 100 was calculated. Conversely, STO cells did not develop ascites. The animals were sacrificed the day after the last CpG-ODN1826 treatment (Fig. 1) and necropsy was performed to observe tumor spread in the abdominal cavity. Solid i.p. masses were gently detached from organs and abdominal wall, removed and weighed. Tumor weight inhibition percentage (TWI %) was used to assess the antitumor activity of CpG-ODN1826. Tumor-free treated mice at the end of experiment were considered cured. Drug treatment toxicity was determined as body weight loss and lethal toxicity.

At the end of the experiment with late-stage STO tumors, peritoneal washings were done by i.p. injection and withdrawal of 5 ml saline using heparinised syringe with 22-gauge needle. The lavage fluid was then transferred into centrifuge tubes and maintained on ice until immunological analysis.

### Flow cytometry and IgM purification

Leukocyte population was evaluated in peritoneal washing and spleen cell suspension obtained from mice bearing late-stage STO tumors, treated or not with CpG-ODN. Briefly, after red blood cells lysis, cells were stained (30 min at 4 °C) with the following antibodies: CD45APCeFluor780 (clone 30-F11; eBioscience, San Diego, CA, USA); CD49bFITC (clone DX5; Miltenyi, Bergisch Gladbach, Germany); CD11bPECy5 (clone M1/70; eBioscience); Ly6G/GR-1PE (clone RB6-8C5; Southern Biotech, Birmingham, AL, USA); F4/80PerCPCy5.5 (clone BM8; eBioscience); CD11cPECy7 (clone N418; eBioscience); CD11bPerCPCy5.5 (clone M1/70; eBioscience); CD5PEVio770 (clone 53-7.3; Miltenyi); CD23FITC (clone B3B4; Miltenyi); CD19APC (clone 6D5, Miltenyi). Rat anti-mouse CD16/CD32 monoclonal antibody (eBiosciences) was used to prevent nonspecific binding to mouse Fc receptors. Cells were examined by FACSCanto flow cytometer (Becton–Dickinson, San Jose, CA, USA) and data analyzed using FlowJo software (TreeStar Inc., Ashland, OR, USA). Analyses were performed gating on CD45 + cells after doublet exclusion.

Moreover, IgM were purified from peritoneal lavages by affinity chromatography on HiTrap IgM Purification HP column (GE HealthCare, Uppsala, Sweden). The concentration of purified IgM was determined by Pierce BCA Protein Assay Kit (Thermo Scientific, Waltham, MA USA).

### Statistical analysis

The percentage of ascites-free mice over time was estimated by the Kaplan–Meier product limit method and compared by the log-rank test. Student’s t test was used to assess differences in tumor weights and immune infiltrate in control versus CpG-ODN1826-treated mice. All tests were two-sided. *P* values <0.05 were considered statistically significant.

## Results

**Fig. 2:**
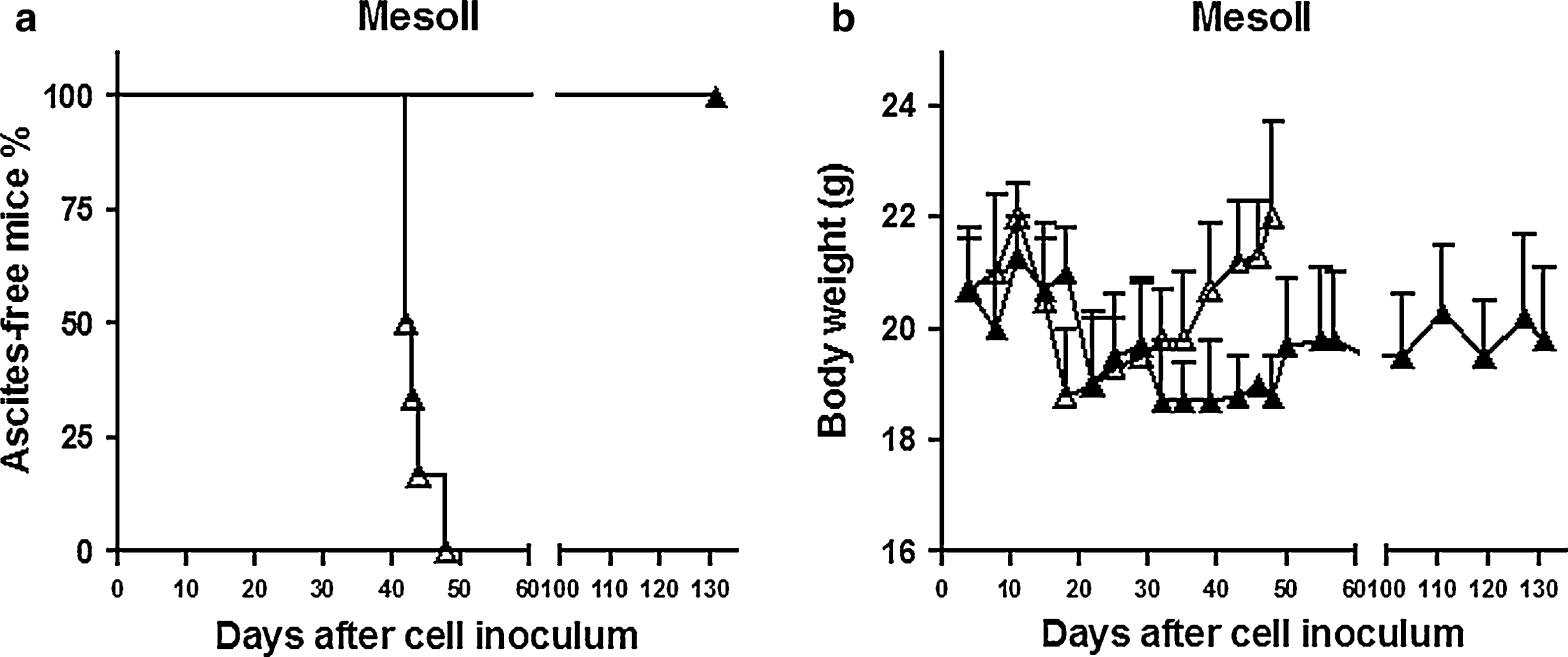
Efficacy of i.p. CpG-ODN1826 (20 µg/mouse, qdx5d/wx4w) against orthotopic DMPM MesoII xenografts. **a** Kaplan–Meier plot of the percentage of ascites-free mice over time since i.p. MesoII cell injection. Mice (six animals/group) were randomized to receive saline (Δ) or CpG-ODN1826 (▲). The treatment started 4 days after cell inoculum (early-stage tumors). **b** Body weight variations in the two experimental groups reported in *panel* (**a**)

The efficacy of CpG-ODN1826 (delivered i.p. qdx5d/wx4w) was initially tested in SCID mice i.p. bearing early-stage (i.e., 4 days after cell inoculum) MesoII tumors. Following i.p. injection of MesoII cells, all control (saline-treated) mice developed ascites in a median time of 42.5 days (range 42–48 days) and underwent increased abdominal volume and body weight (Fig. 2a,b). At necropsy, hemorrhagic effusion, together with a large tumor mass at omentum, was found in all control mice (6/6). In addition, multifocal small nodules, widely scattered on mesentery, diaphragm and abdominal organs, were present. The mean (±SD) volume of removed effusion was 2.05 ± 1.4 ml and the mean (±SD) weight of solid masses was 1460 ± 776 mg (Table 1). Strikingly, 102 days after the last administration of CpG-ODN1826, no treated animal had yet presented ascites (T/C >308 %; *P* = 0.0004) (Fig. 2a). Moreover, no evidence of tumor masses was found at necropsy (Table 1). Body weight loss in treated animals did not exceed 10 % (Fig. 2b) and no toxic death occurred. Thus, CpG-ODN1826 safely cured 100 % mice (6/6), exhibiting impressive efficacy in early-stage MesoII orthotopic xenografts.

**Table 1:** Antitumor effects of i.p. CpG-ODN1826 (20 µg/mouse, qdx5d/wx4w) against early- and late-stage DMPM orthotopic xenografts

Model	Drug	Treatment start (day)	Tumor take^a^	Tumor weight (mg)		TWI %^b^	*P* ^c^
				Median	Mean ± SD		
MesoII	Saline	4	6/6	1360	1460 ± 776		
	CpG-ODN1826		0/6	–	–		
STO	Saline	4	6/6	625	695 ± 206		
	CpG-ODN1826		2/6	0	43 ± 60	94	0.00005
	Saline	11	10/10	996	1120 ± 550		
	CpG-ODN1826		10/10	340	381 ± 99	66	0.0009

MesoII and STO cells (2.5 × 10^7^ and 10^7^/mouse, respectively) were inoculated i.p. in female SCID mice on day 0. Animals were sacrificed at ascites onset (MesoII) or the day after the last CpG-ODN1826 administration (STO); i.p. tumor masses were removed and weighed

^a^Number of mice with i.p. macroscopic tumors out of number of DMPM cell-injected mice

^b^Tumor weight inhibition percentage in treated over control mice

^c^By Student’s t test over saline-treated control mice

**Fig. 3:**
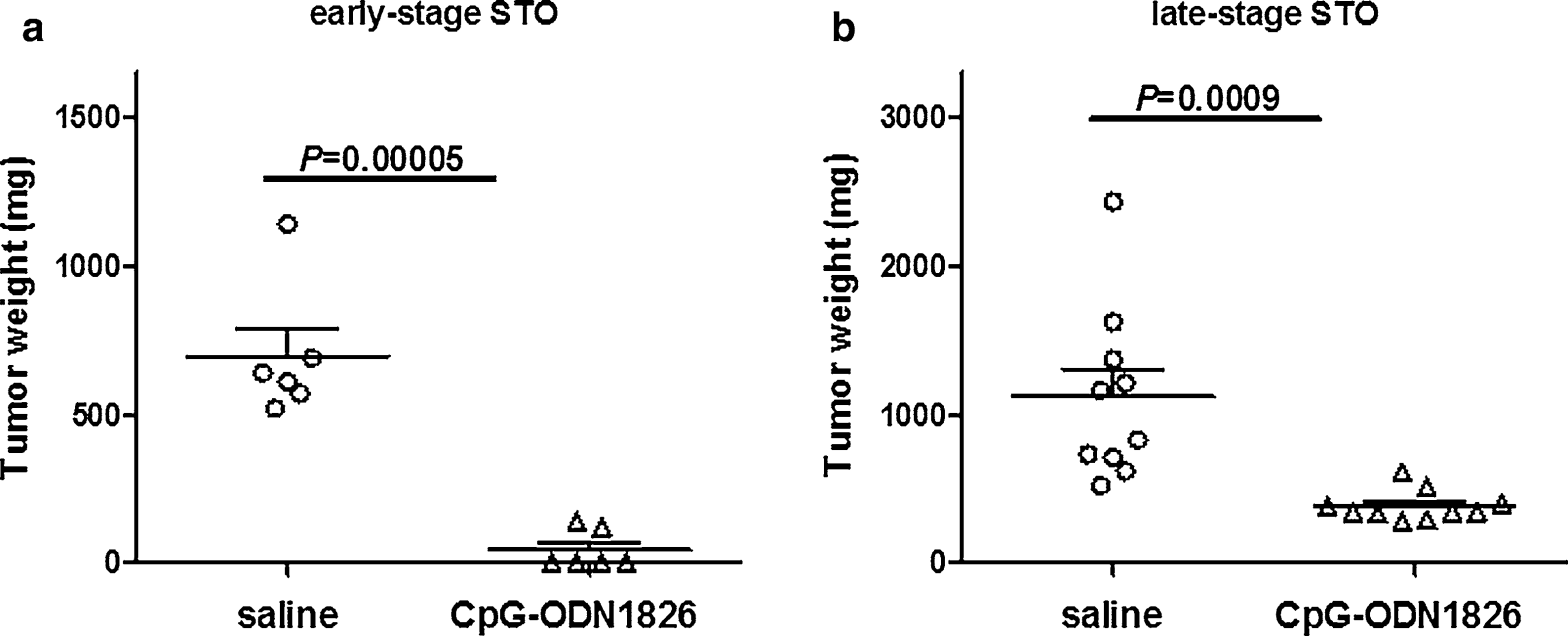
Efficacy of i.p. CpG-ODN1826 (20 µg/mouse, qdx5d/wx4w) against orthotopic DMPM STO xenografts. **a** Orthotopic STO tumor weight distribution. Mice (six animals/group) were randomized to receive saline or CpG-ODN1826. The treatment started 4 days after cell injection (early-stage tumors). **b** Mice (ten animals/group) were randomized to receive saline or CpG-ODN1826. The treatment started 11 days after cell injection (late-stage tumors)

CpG-ODN1826 was then delivered to SCID mice i.p bearing early-stage (4 days after cell inoculum) STO tumors under the same treatment conditions used for MesoII. At day 30 from cell inoculum, i.e. the day after the last CpG-ODN1826 administration, animals were sacrificed. At necropsy, all control mice (6/6) showed considerable tumor growth with the same pattern of distribution of neoplastic masses observed with MesoII. Conversely, the majority of CpG-ODN1826-treated mice (4/6) were macroscopically tumor-free. The mean (±SD) weight of tumor masses was 695 ± 206 mg and 43 ± 60 mg in control and treated mice, respectively, showing 94 % (*P* = 0.00005) drug-induced tumor growth inhibition (Fig. 3a; Table 1). The agent was well tolerated with a maximum body weight loss of 13 % and no toxic death. The data indicate high efficacy of CpG-ODN1826 against early-stage STO orthotopic xenografts.

Based on such excellent results, the antitumor effect of CpG-ODN1826 was tested against the STO model at a late-stage of development (i.e., 11 days after cell inoculum). The day after the last CpG-ODN1826 administration, the animals were sacrificed. At necropsy, i.p. DMPM growth was found in all control and treated mice. However, animals receiving CpG-ODN1826 presented a tumor burden significantly reduced with respect to controls. Specifically, the mean (±SD) weight of tumor masses collected from abdominal cavity was 1120 ± 55 and 381 ± 99 mg (*P* = 0.0009) in control and CpG-ODN1826-treated mice, respectively (Fig. 3b; Table 1), indicating that the agent was able to reduce by 66 % the growth of STO orthotopic xenograft model even when treatment was started at an advanced stage of disease.

**Fig. 4:**
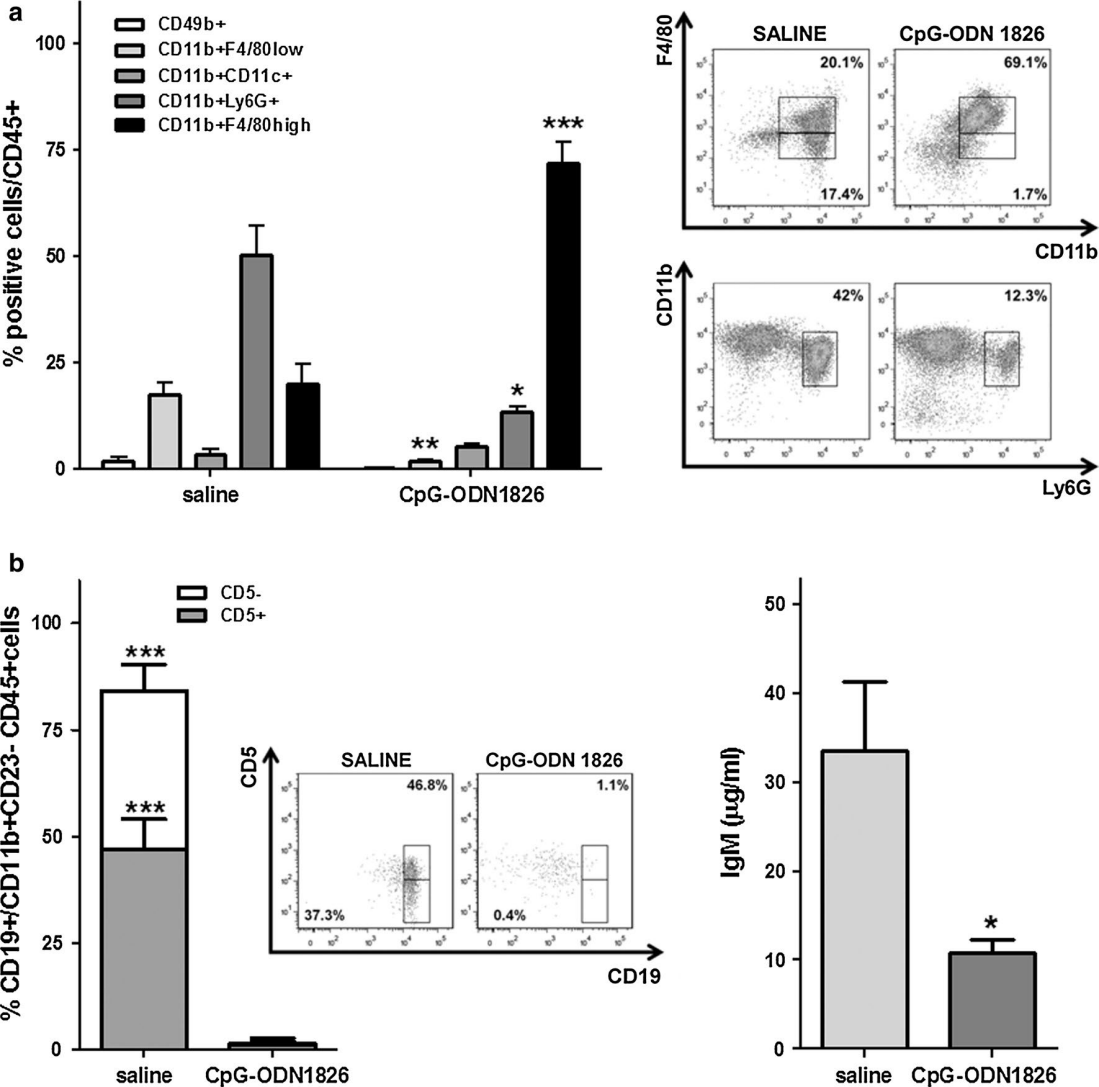
Flow cytometric and affinity chromatography analyses of cellular and humoral peritoneal immunity in CpG-ODN1826 (20 µg/mouse, qdx5d/wx4w) treated late-stage STO tumor-bearing mice. **a** Percentage of NK cells (CD49b+), monocytes/macrophage precursors (CD11b+ F4/80low), DC (CD11b+ CD11c+), granulocytes (CD11b+ Ly6G+), macrophages (CD11b+ F4/80high) among CD45 + cells and dot plots of significantly modulated subpopulations. **b** Percentage and dot plots of B-1 cells (CD19 + gated on CD11b+ CD23- among CD45 + FSClowSSClow cells) expressing or not CD5 (*left*); IgM concentration (*right*). All *histograms* represent pooled data (mean ± SE) from peritoneal lavages of 4 mice/group. *P < 0.05; **P < 0.01; ***P < 0.001

At the end of the experiment, the composition of peritoneal immune cell infiltrate from control and CpG-ODN1826-treated STO-bearing mice (4 animals/group) was analyzed by flow cytometry. Specifically, the percentage of macrophages (CD11b+ F4/80high), granulocytes (CD11b+ Ly6G+), NK (CD49b+) and dendritic cells (DC) (CD11b+ CD11c+) was evaluated among CD45+ gated cells. A strong increase of macrophages (CD11b+ F4/80high) was observed in CpG-ODN1826-treated mice as compared to untreated mice (mean ± SD: 69.1 ± 4.5 % versus 20.1 ± 4.6 %; *P* = 0.0003) (Fig. 4a). In parallel, a reduction of granulocytes (CD11b+ Ly6G+) (mean ± SD: 12.3 ± 1.3 % in CpG-ODN1826-treated versus 42.0 ± 9.7 % in control mice; *P* = 0.023) and of CD11b + F4/80low cells, which include monocytes and/or macrophage precursors, (mean ± SD: 1.7 ± 0.5 % in CpG-ODN1826-treated versus 17.4 ± 2.8 % in untreated mice; *P* = 0.0016) was detected (Fig. 4a). The frequency of NK cells (CD49b+) and DC (CD11b+ CD11c+) was similar in the two experimental groups (Fig. 4a). Flow cytometry analysis was also performed on splenocytes obtained from the same animals. No significant differences in the percentage of monocytes, macrophages, granulocytes, NK cells and DC were observed (data not shown), suggesting that CpG-ODN1826 treatment modified the composition of leukocyte infiltrate only locally.

SCID mice are characterized by the absence, or a marked reduction, of conventional B lymphocytes and by the presence of B-1 cells, a B lymphocyte subpopulation residing in the peritoneal and pleural cavities, which is nearly the only source of natural antibodies (IgM) in these animals [10, 11]. Since CpG-ODN1826 has been demonstrated to interact with B-1 lymphocytes [12, 13], the level of IgM, as a marker of CpG-ODN activity on B-1 cells, was assessed in peritoneal lavages obtained from CpG-ODN1826-treated and control mice. A significant reduction of IgM concentration (*P* = 0.029) was detected in CpG-ODN1826-treated mice as compared to untreated mice (Fig. 4b). To confirm such an unpredicted result, we carried out an additional experiment in late-stage STO bearing mice, under the same treatment conditions, to directly assess the percentage of B-1 cells, together with IgM levels, in peritoneal lavages. Flow cytometry analysis revealed a marked reduction of B-1cells (CD19+ CD11b+ CD23−), expressing or not CD5, and a significant decrease of IgM concentration (11 ± 2.6 µg/ml versus 27.6 ± 3.1 µg/ml, *P* = 0.015) in CpG-ODN1826-treated mice as compared to controls (Fig. 4b).

## Discussion

Since the biology of DMPM is largely unknown, and the cellular and molecular mechanisms responsible for its clinical aggressiveness and relative chemoresistance have not yet been elucidated, the identification of novel therapeutic targets for the design of more efficient treatment approaches remains a main challenge. The need for novel therapeutic strategies is also highlighted by statistical projections of mesothelioma-related deaths, which predict continuing increases in many countries [14], mainly as a late consequence of the widespread use of asbestos. In addition, the lack of DMPM experimental models represents an important obstacle for the preclinical development of novel therapeutics.

In the present study, we assessed the efficacy of CpG-ODN1826 in two DMPM orthotopic xenografts (MesoII and STO), which properly recapitulate the dissemination pattern in the peritoneal cavity of human DMPM [8] and, for this reason, represent valuable models for investigating novel therapeutics. As single agent, CpG-ODN1826 displayed significant antitumor activity against both early- and late-stage DMPM xenograft models. Specifically, CpG-ODN1826, delivered i.p. qdx5/wx4w, completely abolished or markedly impaired take and growth of DMPM models in the abdominal cavity when treatment started a few days after tumor cell inoculum in SCID mice. In addition, in MesoII-bearing mice, CpG-ODN1826 was able to considerably lengthening ascites-free survival. In fact, no sign of ascites was appreciable in treated animals until the end of the experiment (i.e., 102 days after the last CpG-ODN1826 administration). This finding is in trend with the reported ability of the agent to restrain ascites by drainage and prolong the life span of mice bearing established ascites from i.p. ovarian carcinoma xenografts [15, 16]. Angiogenic factors produced by tumors can allow fluid accumulation by increasing the permeability of micro-vessels in the serosa. However, in the i.p. ovarian carcinoma xenograft model, the levels of vascular endothelial growth factor, platelet derived growth factor and basic fibroblast growth factor in i.p. fluid were not affected by CpG-ODN1826 treatment [15], suggesting other possible mechanisms through which the agent inhibits ascites development.

When the therapeutic effects of CpG-ODN1826 were assessed in late-stage STO xenografts, a significant tumor growth impairment was observed, thus indicating that the agent maintains a remarkable antitumor activity even against established tumors, in the presence of an acceptable toxicity. It should be noted that the growth inhibition of human DMPM xenografts was observed in mice injected with a murine specific TLR9 agonist, making unlikely that the observed antitumor activity was ascribable to a direct interaction between CpG-ODN1826 and tumor cells, as different CpG-ODN sequences are required for stimulation of mouse and human TLR9-positive cells.

Flow cytometry analysis of peritoneal infiltrate from late-stage STO-bearing mice revealed a strong increase of macrophage infiltration after CpG-ODN treatment. It is well known that tumor recruited macrophages can support tumor progression [17]; conversely, macrophages activated by microbial products (i.e., TLR ligands and NOD specific agonists) and pro-inflammatory cytokines (i.e., Interferon gamma and Tumor necrosis factor (TNF) alpha) have shown antitumor activity [18]. In this context, macrophages locally activated by CpG-ODN1826 in the peritoneal cavity may promote killing of tumor cells through a variety of mechanisms. Specifically, the antitumor effect can be mediated by the production of cytotoxic and anti-proliferative molecules, such as reactive oxygen and reactive nitrogen intermediates, which can induce DNA damage [19], and TNF superfamily members, which can mediate apoptosis of cells expressing the respective receptors [20, 21]. Moreover, macrophages might indirectly enhance NK-mediated antitumor activity by secreting chemokines, such as CCL5 and CXCL10, and cytokines, such IL-12 and IL-23.

Differences between CpG-ODN1826- and saline-treated mice were also observed through the analysis of IgM in peritoneal lavages, with a strong reduction of IgM abundance in CpG-ODN-treated mice. Such IgM reduction might be related to the ability of CpG-ODN to induce differentiation of peritoneal B-1 cells into myeloid-like phagocytic cells, as recently reported [22]. Accordingly, flow cytometry analysis using a panel of antibodies to detect B-1 cells in peritoneal lavages revealed that this population almost disappeared from peritoneal cavity of mice treated with CpG-ODN1826. However, since the role of B-1 in tumor growth has been poorly investigated, we cannot define whether CpG-ODN1826-induced differentiation of B-1 cells might be involved in DMPM xenograft growth inhibition. Interestingly, recent studies attributed to B-1 cells an important role in tumor progression in a melanoma model, showing that physical contact between B-1 lymphocytes and tumor cells was responsible for changes in the expression of metastasis-associated genes [23, 24].

In summary, the impressive efficacy displayed by CpG-ODN1826 in mice with low tumor burden suggests a possible clinical utility of the agent as local adjuvant therapy in DMPM patients who underwent CRS + HIPEC. In addition, the finding that CpG-ODN1826 maintains antitumor activity also against late-stage DMPM xenografts, together with previous evidence indicating that the agent synergizes with several antitumor drugs, either cytotoxic (gemcitabine, camptothecins and cisplatin) [25] or target-specific (cetuximab) [16], highlights the possibility that CpG-ODN1826 could have a role in combined strategies for treating patients who are not eligible for CRS +HIPEC.

## Conclusions

DMPM is a rapidly fatal tumor with scanty therapeutic options. Here we demonstrate for the first time that locally administered CpG-ODN1826, a synthetic DNA sequence recognized by TLR9 and able to induce innate/adaptive immune response, displays significant antitumor activity against early- and late-stage DMPM orthotopic xenograft models, in the absence of severe toxicity. These findings support the possible clinical relevance of a novel CpG-ODN1826-based immunotherapy approach for the disease. In this context, a deeper understanding of the determinants of CpG-ODN1826 activity will be of paramount importance for the definition of biomarkers for the selection of patients more likely to respond and maximize treatment benefits.

## Abbreviations

CRS: cytoreductive surgery
DC: dendritic cells
DMPM: diffuse malignant peritoneal mesothelioma
HIPEC: hyperthermic i.p. chemotherapy
i.p.: intra-peritoneal
NK: natural killer
ODN: oligodeoxynucleotide
SCID: severe combined immunodeficiency
TLR: toll-like receptors
TNF: tumor necrosis factor
TWI %: tumor weight inhibition percentage
